# Prevalence of methemoglobinemia after lidocaine as add-on therapy in neonatal seizures

**DOI:** 10.1007/s00431-026-06958-8

**Published:** 2026-04-28

**Authors:** Bregje O. van Oldenmark, Mathies Rondagh, Kirsten J. M. Schimmel, Arjan B. te Pas, Enrico Lopriore, Linda S. de Vries, Sylke J. Steggerda

**Affiliations:** 1 Willem-Alexander Children’s Hospital, Department of Pediatrics, Division of Neonatology, Leiden University Medical Center, Albinusdreef 2, 2300RC Leiden, South-Holland The Netherlands; 2 Department of Clinical Pharmacy and Toxicology, Leiden University Medical Center, Albinusdreef 2, 2300RC Leiden, South-Holland The Netherlands

**Keywords:** Lidocaine, Methemoglobinemia, Neonatal Seizures, Newborns, Seizure management

## Abstract

Methemoglobinemia is a rare but potentially serious complication of lidocaine (LDC) use for local anesthesia in infants and young children, yet its prevalence during continuous systemic infusion for neonatal seizures remains unclear. Given the increasing use of LDC as add-on therapy for neonatal seizures, this retrospective cohort study evaluated methemoglobin (MetHb) levels in 51 neonates with (serial) measurements within 96 h before and after LDC infusion. Methemoglobinemia was defined as MetHb > 2% in arterial or capillary blood gas samples. Prevalence increased from 6% (3/51) before LDC to 47% (24/51) after administration (*p* < 0.001). Most neonates had mildly elevated MetHb levels (< 5%); three had a MetHb level > 5%, with a highest peak of 9.5%. In one neonate LDC was discontinued due to hypoxemia, the others were asymptomatic. In neonates who developed methemoglobinemia after LDC, the mean time to peak MetHb was 29.1 h (95% CI 11.1–73.3), and mean time to recover to < 2% after the peak was 27.5 h (95% CI 8.4–75.5). Over half of neonates receiving regimen III (> 36 weeks without therapeutic hypothermia [TH]) developed methemoglobinemia, compared to none on regimen IV (with TH). This suggests an association with a higher cumulative dose and longer infusion duration.

*Conclusion*: In conclusion, methemoglobinemia occurred in nearly half of neonates receiving continuous LDC for seizures and was associated with the dosing regimen. It is important to be aware of this complication, especially in infants who develop respiratory deterioration and oxygenation problems following continuous LDC administration.

**Supplementary Information:**

The online version contains supplementary material available at 10.1007/s00431-026-06958-8.

**Table:** 

**What is Known:** • *Methemoglobinemia (MetHb > 2%) is a rare but potentially serious complication of lidocaine used for local anesthesia in infants and young children*. • *Data on its prevalence during continuous lidocaine infusion for neonatal seizures is currently lacking*.		
**What is New:** • *Methemoglobinemia occurs in approximately half of the neonates with continuous lidocaine infusion for neonatal seizures*. • *The risk depends on the dosing regimen as it is associated with a longer duration and higher cumulative dose of lidocaine infusion*.		

## Introduction

Infants are at increased risk of developing drug-induced methemoglobinemia and several reports have described this following local anesthetic administration of lidocaine (LDC) [1–5]. Nowadays, systemic LDC infusion is increasingly used as add-on therapy for neonatal seizures and has shown high efficacy, with reported response rates ranging from 60 to 92% [6, 7]. Up to now, one case report described severe symptomatic methemoglobinemia associated with prolonged LDC use for the treatment of neonatal seizures [2].

LDC is metabolized to oxidizing compounds that convert hemoglobin iron from its ferrous (Fe^2⁺^) to ferric (Fe^3^⁺) state, forming methemoglobin (MetHb), which cannot bind oxygen and reduces tissue oxygen delivery, potentially causing hypoxemia and metabolic acidosis [1, 2]. Neonates are particularly vulnerable because their methemoglobin-reductase system is physiologically immature. A MetHb proportion of 0–2% is considered normal, but when it is 10–15%, clinical symptoms, such as cyanosis, numbness and feeding intolerance may occur [1, 2]. MetHb levels > 70% may be lethal. Management of methemoglobinemia includes supplemental oxygen, and intravenous methylene blue is recommended for symptomatic neonates or when MetHb exceeds 20% [1]. However, clinicians should use methylene blue with caution, as it may paradoxically worsen methemoglobinemia in neonates, particularly in those with glucose-6-phosphate dehydrogenase deficiency or at higher doses [8].

Despite the increasing use of LDC as add-on therapy for neonatal seizures, the prevalence and clinical implications of methemoglobinemia have not been described. In our neonatal intensive care unit, MetHb levels are routinely monitored in blood gas samples, enabling longitudinal assessment in neonates treated with LDC. This study was aimed at determining the prevalence of methemoglobinemia in neonates receiving LDC for seizures and at evaluating methemoglobin levels before, during, and after LDC infusion.

## Methods

### Study design, setting, and participants

This retrospective single-center cohort study was conducted at the Leiden University Medical Center (LUMC, the Netherlands), with institutional review board approval (Medical Research Involving Human Subjects Act, ref. 23–3117). The study adhered to the Strengthening the Reporting of Observational Studies in Epidemiology (STROBE) reporting guideline (Supplementary Material 1). Neonates born between 2015 and 2025, who received intravenous LDC as treatment for neonatal seizures were eligible if MetHb levels from serial blood gas analyses before, during, and after LDC administration were available. Neonates without or only a single MetHb measurement were excluded. Patients were identified via pharmacy records and verified in electronic files.

### Lidocaine dose regimen

All neonates received a 2 mg/kg LDC loading dose over 10 min. Subsequent dosing was applied according to national guidelines and varied by weight, gestational age, and therapeutic hypothermia (TH): (regimen I) preterm < 36 weeks (0.8–1.5 kg): 5 mg/kg/h for 4 h, 2.5 mg/kg/h for 6 h, and 1.25 mg/kg/h for 12 h; (regimen II) preterm < 36 weeks (1.5–2.5 kg): 6 mg/kg/h for 4 h, 3 mg/kg/h for 6 h, and 1.5 mg/kg/h for 12 h; (regimen III) neonates ≥ 36 weeks (≥ 2.5 kg): 6 mg/kg/h for 6 h, 4 mg/kg/h for 12 h, and 2 mg/kg/h for 12 h; and (regimen IV) neonates ≥ 36 weeks undergoing TH: 4 mg/kg/h for 6 h followed by 2 mg/kg/h for 12 h.

### Clinical characteristics

MetHb was measured routinely when performing blood gas analysis, by co-oximetry (RAPID Point 500 Systems, Siemens, and ABL90 Flex, Radiometer), using arterial or capillary samples from 96 h before to 96 h after LDC administration. Methemoglobinemia was defined as MetHb > 2% of total hemoglobin, in accordance with neonatal reference ranges and prior published studies [2]. Demographic and clinical data included gestational age, birth weight, sex, mode of delivery, Apgar scores, persistent pulmonary hypertension of the newborn (PPHN) treated with nitric oxide (NO), sepsis, meningitis, TH for hypoxic-ischemic encephalopathy, and mortality.

### Statistical analysis

Continuous data are presented as means with 95% confidence intervals (95% CI) or medians with interquartile ranges (IQR), and categorical data as counts and percentages. Peak MetHb was defined as maximum measured MetHb level; recovery time as maximum-to-baseline (MetHb < 2%). For neonates who developed methemoglobinemia under LDC treatment, peak MetHb time was the interval from LDC start to the highest level, and recovery time from peak to baseline < 2%. Group-level MetHb trajectories were derived from 6-h epochs; mean MetHb and 95% CIs were calculated per epoch. Analyses were performed in SPSS v29 (IBM) and MATLAB v2022b (MathWorks).

## Results

A total of 87 neonates received intravenous LDC. After excluding 2 without a documented LDC administration time and 34 without available serial MetHb measurements, 51 neonates were included for final analysis. Patient characteristics and dosing regimens are shown in Table 1.

**Table 1:** Clinical characteristics

Clinical characteristics	Total study population (*n* = 51)	Neonates with methemoglobinemia (*n* = 24)	Neonates without methemoglobinemia (*n* = 27)
Gestational age, *weeks.days*, median (IQR)	38.1 (37.1–39.1)	40.0 (38.1–40.6)	38.3 (34.4–40.3)*
Preterm (GA < 36 weeks), n (%)	10 (20)	1 (4)	8 (30)
Female, *n* (%)	26 (50)	11 (46)	15 (56)
Birthweight, *grams*, mean (95% CI)	3385 (2734–3800)	2580 (3309–3850)	2870 (2478–3262)
Apgar score, Median (IQR)			
5 min	7 (3–9)^†^	9 (1–8)	4 (1–8)^§^
10 min	9 (5–9)^‡^	9 (7–10)	6 (2–9)
Seizure etiology, *n* (%)			
HIE	21 (41)	2 (13)	19 (70)
Arterial ischemic stroke	6 (12)	5 (21)	1 (4)
Hemorrhagic stroke	8 (16)	7 (29)	1 (4)
CNS infection	7 (15)	4 (17)	3 (11)
Metabolic disorder	4 (8)	3 (13)	1 (4)
Genetic condition	4 (8)	2 (8)	2 (7)
LDC regimen, *n* (%)			
I (22 h, 49.25 mg/kg)	3 (6)	0 (0)	3 (11)
II (22 h, 62 mg/kg)	5 (10)	3 (13)	2 (7)
III (30 h, 110 mg/kg)	32 (63)	21 (87)	11 (41)
IV (18 h, 50 mg/kg)	11 (21)	0 (0)	11 (41)
Therapeutic hypothermia, *n* (%)	11 (52)^||^	0 (0)	11 (41)
PPHN for which NO was used, *n* (%)	8 (16)	5 (21)	3 (11)
Sepsis, *n* (%)	4 (6)	1 (4)	3 (11)
Neonatal death, *n* (%)	29 (40)	7 (29)	21 (78)

^*^1 infant missing: term pregnancy (exact GA unknown)

^†^4 neonates missing

^‡^6 neonates missing

^§^1 infant missing

^||^Among 21 neonates with HIE

**Fig. 1:**
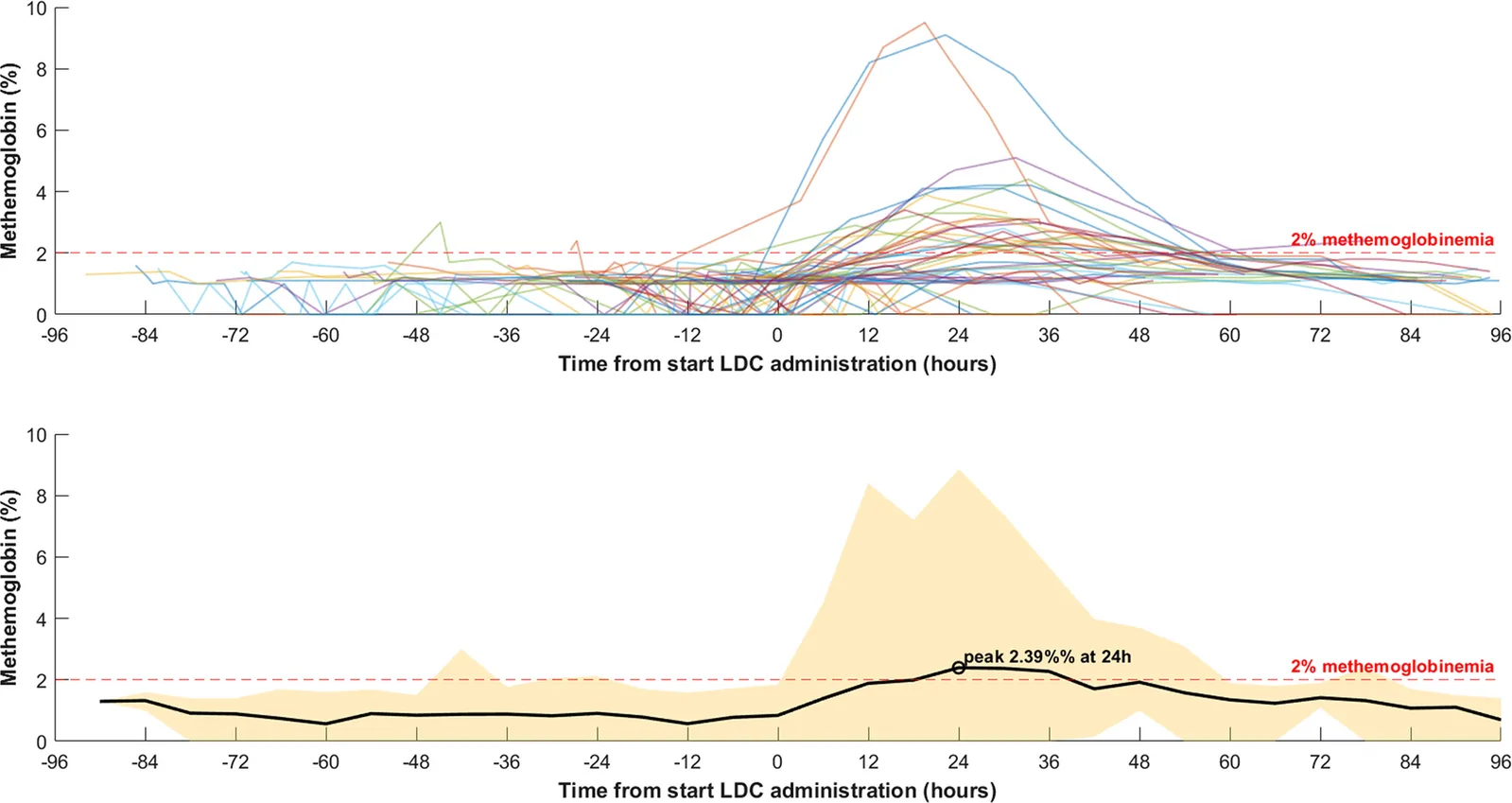
Methemoglobin levels before, during and after LDC treatments for all individuals and the total study population. Upper panel: individual patient MetHb measurements are plotted relative to the start of LDC administration (*t* = 0). Each line represents a single patient. The red dashed line marks the clinical threshold for methemoglobinemia (2%). Lower panel: group-level MetHb measurements are shown as mean levels in 6-h epochs with corresponding 95% confidence intervals (shaded band)

Within the 192-h observation window, 50% (27/51) of neonates show at least one MetHb level > 2% (Fig. 1; Table 1). Methemoglobinemia prior to the start of LDC occurred in 6% (3/51) of neonates, whereas 47% (24/51) developed methemoglobinemia after the start of LDC administration (*p* < 0.001). Among neonates who developed methemoglobinemia after LDC, the mean peak MetHb level occurred at 29.1 h (95% CI 11.1–73.3) and the mean time to recovery to a baseline < 2% was 27.5 h (95% CI 8.4–75.5) after reaching the peak of MetHb. Overall, the mean peak of MetHb for the total population was 2.4% and was reached at 24 h. Three neonates had a MetHb level > 5%, with highest peak at 5.1%, 9.1% and 9.5%, respectively.

Most neonates receive regimen III (32/51; 63%), followed by regimen IV (11/51; 22%), regimen II (5/51; 10%) and regimen I (3/51; 6%) (Table 1). Among neonates with regimen IV, none developed methemoglobinemia. In contrast, methemoglobinemia occurred in 20% (1/5) of neonates receiving regimen II and in 72% (23/32) of those receiving regimen III. Among neonates in regimen III, the median time to peak level was 30 h (95% CI, 10–78 h), and the median time to recovery to baseline was 29 h (95% CI, 8–76 h).

In one infant LDC was discontinued after 21 h due to an increase in MetHb levels from 1.1% before start to 3.7%, 8.9% and finally 9.4% during administration. During this period the infant was stable on mechanical ventilation but developed an increased oxygen requirement from 21 to 30%. The other infants with methemoglobinemia showed no hypoxemia and required no clinically significant adjustments in respiratory support attributable to methemoglobinemia.

## Discussion

### Key results

In our study population, approximately half of neonates receiving continuous LDC infusion for neonatal seizures developed methemoglobinemia, and this was strongly associated with dosing regimen. Overall, MetHb values were mildly increased after LDC and infants were predominantly asymptomatic. LDC was discontinued in only one patient because of hypoxemia. Given the high prevalence of mostly mild methemoglobinemia in neonates receiving continuous LDC infusion, clinicians should be aware of this potential complication, particularly in infants receiving prolonged treatment. In case of unexplained respiratory deterioration or hypoxemia during LDC therapy, measuring methemoglobin levels is recommended.

### Result in the context of what is known

The occurrence of methemoglobinemia was associated with the dosing regimen. None of the neonates with regimen IV (in the case of TH) developed methemoglobinemia, likely reflecting the lower cumulative dose and shorter infusion duration used in this group. In contrast, more than half of neonates who received dose regimen III (> 36 weeks without TH) developed methemoglobinemia. Together, these findings suggest that a longer infusion duration (30 vs. 18 h) with a higher cumulative dose (110 vs. 50 mg/kg) is a key contributor to the development of methemoglobinemia.

The fact that we use different dosing regimens for preterm infants, full-term infants with HIE and TH and full-term infants without TH probably explains the differences in baseline characteristics and mortality between infants with and without methemoglobinemia.

A previous case report described severe methemoglobinemia during LDC treatment for neonatal seizures, with MetHb levels up to 14%, leading to hypoxemia and cyanosis that required increased oxygen and CPAP; MetHb levels declined and ventilatory support was weaned after LDC discontinuation [2]. Additional neonatal cases have been reported after maternal LDC during delivery, LDC prilocaine (EMLA) cream, and following subcutaneous LDC for surgical procedures [4, 5, 9]. Neonates are particularly vulnerable to drug-induced methemoglobinemia due to several reasons [3]. First, fetal hemoglobin, is more easily oxidized to MetHb compared with adult hemoglobin [3]. Second, the activity of the NADH-dependent MetHb reductase pathway, the primary mechanism for reducing MetHb back to hemoglobin, is reduced in neonates [3]. Third, neonates have a higher total body water content and lower plasma protein binding capacity, resulting in a larger free fraction of LDC and other oxidizing agents [10]. In addition, LDC is primarily bound to alpha-1 acid glycoprotein, an acute-phase protein, whose concentrations may increase in response to physiological stress and systemic inflammation, such as perinatal asphyxia, and have been shown to approximately double within 48 h of major surgery in neonates [11]. Elevated AAG levels may increase protein binding of lidocaine and thereby reduce the free drug fraction. Interestingly, neonates in our cohort who did not develop methemoglobinemia had lower Apgar scores, higher rates of sepsis and HIE, and higher mortality, suggesting a more severely ill population in whom elevated AAG could potentially have influenced lidocaine protein binding and methemoglobinemia risk [12, 13]. Finally, common perinatal stressors such as hypoxia, sepsis, or acidosis can impair the ability to compensate for elevated MetHb levels, thereby exacerbating clinical toxicity at doses that might be well tolerated in older children or adults [3].

### Strength and limitations

This is the first study reporting on the prevalence and time course of methemoglobinemia before, during, and after LDC treatment for neonatal seizures in a relatively large cohort. First, due to the retrospective design, methemoglobin measurements were not performed according to a standardized protocol but were obtained as part of routine clinical care, which may have introduced measurement bias. It may have resulted in inconsistent timing of methemoglobin measurements, potentially leading to underestimation of peak methemoglobin levels or recovery times. Second, differences in dosing regimens and underlying clinical conditions between patient groups may have introduced confounding and limited causal interpretation of the association between lidocaine dosing and methemoglobinemia. Finally, we did not measure lidocaine plasma concentrations, which limits pharmacokinetic interpretation.

## Conclusion

Methemoglobinemia is a common finding during continuous LDC add-on therapy for neonatal seizures. These findings underscore the importance of clinician awareness, particularly with higher doses or longer infusion duration, and support consideration of MetHb measurement in case of respiratory deterioration with hypoxemia during LDC therapy.

## Supplementary Information

Below is the link to the electronic supplementary material.

ESM 1 (DOCX 33.4 KB)

## Data Availability

All data generated or analyzed during this study are included in this article. Further enquiries can be directed to the corresponding author.
