# Correction to: Prevalence of methemoglobinemia after lidocaine as add-on therapy in neonatal seizures

**DOI:** 10.1007/s00431-026-07337-z

**Published:** 2026-08-26

**Authors:** Bregje O. van Oldenmark, Mathies Rondagh, Kirsten J. M. Schimmel, Arjan B.te Pas, Enrico Lopriore, Linda S. de Vries, Sylke J. Steggerda

**Affiliations:** 1 Department of Pediatrics, Division of Neonatology, Willem-Alexander Children’s Hospital, Leiden University Medical Center, Albinusdreef 2, 2300RC Leiden, South-Holland The Netherlands; 2 Department of Clinical Pharmacy and Toxicology, Leiden University Medical Center, Albinusdreef 2, 2300RC Leiden, South-Holland The Netherlands

**Correction to: European Journal of Pediatrics (2026) 185:314**

**https://doi.org/10.1007/s00431-026-06958-8**

In Table 1 of the original publication, errors were identified.

## Published form:

**Table:** **Table 1** Clinical characteristics

Clinical characteristics	Total study population (*n* = 51)	Neonates with methemoglobinemia (*n* = 24)	Neonates without methemoglobinemia (*n* = 27)
Gestational age, *weeks.days*, median (IQR)	38.1 (37.1–39.1)	40.0 (38.1–40.6)	38.3 (34.4–40.3)^*^
Preterm (GA < 36 weeks), n (%)	10 (20)	1 (4)	8 (30)
Female, *n* (%)	26 (50)	11 (46)	15 (56)
Birthweight, *grams*, mean (95% CI)	3385 (2734–3800)	2580 (3309–3850)	2870 (2478–3262)
Apgar score, Median (IQR)			
5 min	7 (3–9)^†^	9 (1–8)	4 (1–8)^§^
10 min	9 (5–9)^‡^	9 (7–10)	6 (2–9)
Seizure etiology, *n* (%)			
HIE	21 (41)	2 (13)	19 (70)
Arterial ischemic stroke	6 (12)	5 (21)	1 (4)
Hemorrhagic stroke	8 (16)	7 (29)	1 (4)
CNS infection	7 (15)	4 (17)	3 (11)
Metabolic disorder	4 (8)	3 (13)	1 (4)
Genetic condition	4 (8)	2 (8)	2 (7)
LDC regimen, *n* (%)			
I (22 h, 49.25 mg/kg)	3 (6)	0 (0)	3 (11)
II (22 h, 62 mg/kg)	2 (4)	3 (13)	2 (7)
III (30 h, 110 mg/kg)	35 (69)	21 (87)	11 (41)
IV (18 h, 50 mg/kg)	11 (21)	0 (0)	11 (41)
Therapeutic hypothermia, *n* (%)	11 (52)^||^	0 (0)	11 (41)
PPHN for which NO was used, *n* (%)	8 (16)	5 (21)	3 (11)
Sepsis, *n* (%)	4 (6)	1 (4)	3 (11)
Neonatal death, *n* (%)	29 (40)	7 (29)	21 (78)

^*^1 infant missing: term pregnancy (exact GA unknown)

^†^4 neonates missing

^‡^6 neonates missing

^§^1 infant missing

^||^Among 21 neonates with HIE

**Corrected form:**

**Table:** **Table 1** Clinical characteristics

Clinical characteristics	Total study population (*n* = 51)	Neonates with methemoglobinemia (*n* = 24)	Neonates without methemoglobinemia (*n* = 27)
Gestational age, *weeks.days*, median (IQR)	38.1 (37.1–39.1)	40.0 (38.1–40.6)	38.3 (34.4–40.3)^*^
Preterm (GA < 36 weeks), n (%)	10 (20)	1 (4)	8 (30)
Female, *n* (%)	26 (50)	11 (46)	15 (56)
Birthweight, *grams*, mean (95% CI)	3385 (2734–3800)	2580 (3309–3850)	2870 (2478–3262)
Apgar score, Median (IQR)			
5 min	7 (3–9)^†^	9 (1–8)	4 (1–8)^§^
10 min	9 (5–9)^‡^	9 (7–10)	6 (2–9)
Seizure etiology, *n* (%)			
HIE	21 (41)	2 (13)	19 (70)
Arterial ischemic stroke	6 (12)	5 (21)	1 (4)
Hemorrhagic stroke	8 (16)	7 (29)	1 (4)
CNS infection	7 (15)	4 (17)	3 (11)
Metabolic disorder	4 (8)	3 (13)	1 (4)
Genetic condition	4 (8)	2 (8)	2 (7)
LDC regimen, *n* (%)			
I (22 h, 49.25 mg/kg)	3 (6)	0 (0)	3 (11)
II (22 h, 62 mg/kg)	5 (10)	3 (13)	2 (7)
III (30 h, 110 mg/kg)	32 (63)	21 (87)	11 (41)
IV (18 h, 50 mg/kg)	11 (21)	0 (0)	11 (41)
Therapeutic hypothermia, *n* (%)	11 (52)^||^	0 (0)	11 (41)
PPHN for which NO was used, *n* (%)	8 (16)	5 (21)	3 (11)
Sepsis, *n* (%)	4 (6)	1 (4)	3 (11)
Neonatal death, *n* (%)	29 (40)	7 (29)	21 (78)

^*^1 infant missing: term pregnancy (exact GA unknown)

^†^4 neonates missing

^‡^6 neonates missing

^§^1 infant missing

^||^Among 21 neonates with HIE

The original article has been corrected.

